# Distributed ISAR Subimage Fusion of Nonuniform Rotating Target Based on Matching Fourier Transform

**DOI:** 10.3390/s18061806

**Published:** 2018-06-04

**Authors:** Yuanyuan Li, Yaowen Fu, Wenpeng Zhang

**Affiliations:** College of Electronic Science, National University of Defense Technology, Changsha 410073, China; fuyaowen@nudt.edu.cn (Y.F.); zhangwenpeng@hotmail.com (W.Z.)

**Keywords:** distributed ISAR, image fusion, matching fourier transform, nonuniform rotation target, maneuvering target

## Abstract

In real applications, the image quality of the conventional monostatic Inverse Synthetic Aperture Radar (ISAR) for the maneuvering target is subject to the strong fluctuation of Radar Cross Section (RCS), as the target aspect varies enormously. Meanwhile, the maneuvering target introduces nonuniform rotation after translation motion compensation which degrades the imaging performance of the conventional Fourier Transform (FT)-based method in the cross-range dimension. In this paper, a method which combines the distributed ISAR technique and the Matching Fourier Transform (MFT) is proposed to overcome these problems. Firstly, according to the characteristics of the distributed ISAR, the multiple channel echoes of the nonuniform rotation target from different observation angles can be acquired. Then, by applying the MFT to the echo of each channel, the defocused problem of nonuniform rotation target which is inevitable by using the FT-based imaging method can be avoided. Finally, after preprocessing, scaling and rotation of all subimages, the noncoherent fusion image containing all the RCS information in all channels can be obtained. The accumulation coefficients of all subimages are calculated adaptively according to the their image qualities. Simulation and experimental data are used to validate the effectiveness of the proposed approach, and fusion image with improved recognizability can be obtained. Therefore, by using the distributed ISAR technique and MFT, subimages of high-maneuvering target from different observation angles can be obtained. Meanwhile, by employing the adaptive subimage fusion method, the RCS fluctuation can be alleviated and more recognizable final image can be obtained.

## 1. Introduction

Conventional monostatic ISAR images are obtained under the condition that the change of the radar observation angle is small (<$5^{{}^{\circ}}$) [1]. For modern high maneuvering aircraft with high speed, stealth and other characteristics, even a slight change of the observation angle can cause a fluctuation of 10 to 15 dB on the RCS [2]. The strong fluctuation of the RCS will cause a deterioration in the ISAR image quality. Therefore, the imaging quality of monostatic ISAR for a high-maneuvering target is easily influenced by the observation angle. Meanwhile, after translation motion compensation, the nonuniform rotation caused by the maneuvering motion will degrade the imaging performance of the conventional FT-based method.

Unlike the conventional monostatic ISAR, the distributed ISAR technique can utilize the data acquired from multiple observation angle to improve the image quality [3,4,5,6]. Each radar sensor is characterized by either transmitting capability or receiving capability. Moreover, the receiving sensors can receive and separate all the transmitting signals. Therefore, any transmitting sensor and any receiving sensor can form a transmitting/receiving channel (or can be considered to form an equivalent self-transmitting and self-receiving sensor). With an appropriate formation of the radar sensors, the target can be observed from multiple observation angles, which provides the ways to overcome the RCS fluctuation.

Recently, the research on the distributed ISAR is making rapid growth. Pastina et al. [3,4,5,6] analyzed the potential of the distributed ISAR to increase the cross-range resolution by exploiting multiple equivalent sensors to increase the global variation of the view angle. However, its effectiveness relies on the assumption that the change of the observation angle is small so that the range-compressed echoes of different channels have stable phases. However, in order to get the target image that overcomes the RCS fluctuation, the observation angles must be very different and the phase stability cannot be guaranteed. Thus, the proposed methods in [3,4,5] are not easy to apply in this scenario. Furthermore, for the high-maneuvering target which is inevitable in real scenarios, there is a nonuniform rotation after translation motion compensation. As a result, the change of observation angle of each equivalent sensor is no longer a linear function with respect to the slow time, which means that the combined view angle is not continuous and cannot provide better resolution. Thus, the methods proposed in [3,4,5] have some limitation for radar formations involving large variation among the individual perspectives and high-maneuvering targets.

Image fusion using subimages obtained from the distributed ISAR system is a solution to overcome the RCS flucation problem of the high maneuvering target. The distributed ISAR system enables the target to be observed from multiple perspectives, and the image fusion instead of echo fusion can reduce the limitation of the change of observation angle. References [7,8] acquire the fusion image after estimating the rotation rate and the bistatic angle from two subimages. Without more observation channels and more RCS information, the improvement of the image quality of the bistatic ISAR is limited. Reference [9] proposed a image fusion method for the uniform rotating target via distributed ISAR, but without consideration for the nonuniform rotating target.

In this work, the authors are interested in the potential of the distributed ISAR to acquire the image of the high-maneuvering target. Thus, the two aforementioned key problems must be considered, namely RCS fluctuation in different observation angles and target nonuniform rotation after translation compensation. For the first problem, the distributed ISAR technique is applied to obtain subimages from different observation angles with different RCS values. Then, the fusion image which contains all the RCS information and with improved quality can be acquired by the non-coherent fusion method.

For the second problem, it is proved that the radar echos can be modelled as a polynomial phase signal (PPS), and the Fourier transform is inappropriate for the azimuth focusing. To solve this problem, the Range-Instantaneous-Doppler (RID) algorithm has been proposed to improve the ISAR image quality, where the Fourier transform is substituted by the time-frequency representations (TFRs). The first approach of RID algorithm is based on the (TFRs) with high concentration and reduced cross-terms, such as the imaging methods based on Short Time Fourier Transform (STFT) [10], the Wigner-Ville distribution (WVD) [11] and so on. Though the RID algorithms performs well in terms of the computational efficiency, they still suffer from the tradeoff between the time frequency concentration and the cross-terms. Also, the obtained RID images are not very stable which will cause diffculty to the subimage fusion step. The second approach is based on the parameters estimation technique, the high quality ISAR images can be obtained by the parameters estimation of the coefficients of the PPS. The parameterized imaging methods (such as [12,13]) are effective for the enhancement of ISAR image. However, for the distributed ISAR system, it is very computational expensive to estimate the signal parameters of all range bins in echoes from all observation channels. However, the rotational parameters of the target can be regarded as invariable in the whole observation time, and the ratio of the rotational acceleration to rotational speed is also fixed. Therefore, estimating the ratio of rotational parameters in one or several range bins, rather than estimating signal parameters in all range bins, may greatly reduce the amount of computation. Wu [14] describes the rotational nonuniformity-relative angular acceleration (RAA) and relative angular jerk (RAJ), and with the estimated RAA and RAJ, rotational nonuniformity compensation is carried out. This method is effective to get the high quality ISAR image, but for the distributed ISAR, it is still cumbersome to construct compensation matrix for each equivalent sensor imaging. Thus, the MFT imaging method is used here. By estimating the rotation parameters, the ISAR images of all observation channels can be obtained directly through MFT.

This paper is organized as follows. After presenting the signal model of distributed ISAR in Section 2, the imaging method of the nonuniform rotation target based on MFT is introduced in Section 3. Then, both simulation and experimental results are presented to validate the effectiveness of the proposed method in Section 4. Finally, we conclude this paper in Section 5.

## 2. Distributed ISAR Echo Model

**Figure 1:**
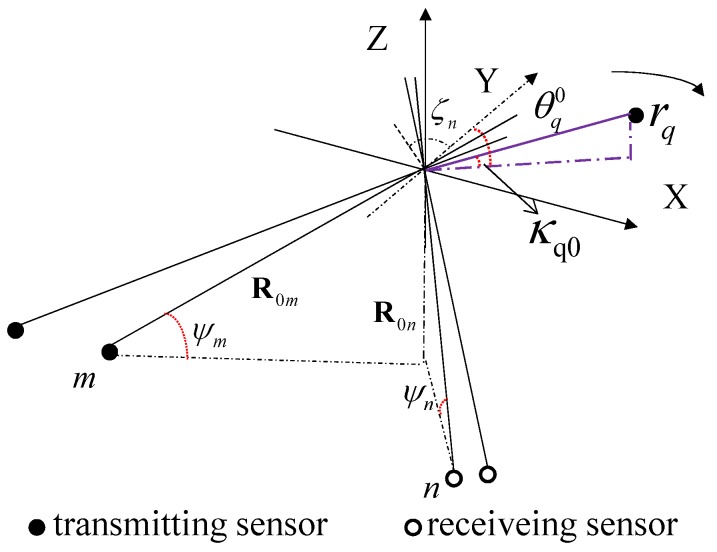
Geometry of the distributed ISAR.

Consider a 3D coordinate system (O,X,Y,Z) with the origin in the target’s fulcrum, and the target’s motion can be decomposed into a translation of the fulcrum and a rotation of the target body. We assume here that any relative translation motion between the distributed sensors and the target fulcrum have been already compensated. Therefore, we can focus on the target rotation. To simply the processing algorithm, the dominant rotation around the vertical axis is considered only. Also, we model the target as a rigid body consisting of *Q* scatterers. The radar formation and the target rotation are shown in Figure 1.

The distributed ISAR system consists of *M* transmitting sensors and *N* receiving sensors as shown in Figure 1. *m* is used to represent the *m*th transmitting sensor and *n* is used to represent the *n*th receiving sensor. Each of them can placed in the ground and carry an antenna appropriately steered toward the moving target in the air. Also, another possible application is that each radar sensor is carried by an aircraft to observe the target on the ground. The detailed placement of the sensors will be introduced later.

Assume the *M* transmitting signals $s_{m}\left(\hat{t}\right)$ are orthogonal, and each receiving sensor has the ability to receive and separate the signals from different transmitting sensors. Thus, I=MN transmitting/receiving channels can be formed. Meanwhile, we assume that all sensors have achieved time synchronization to ensure accurate matching of the transmitting signal and receiving signal. After demodulation and range compression, the received backscattered signal in the *m-n*th observation channel is denoted as

(1) $$S_{mn}\left(\hat{t},t_{p}\right)=\;\sum_{q=1}^{Q}\sigma_{mn,q}p_{m}(\hat{t}-\;\frac{R_{mn,q}\left(t_{p}\right)}{c})e^{-j\frac{2\pi }{\lambda }R_{mn,\;q}\left(t_{p}\right)},$$

where $\hat{t}$ is the fast time, $t_{p}$ is the slow time, *c* is the wave velocity, λ is the carrier wavelength, $\sigma_{mn,q}$ is the scattering coefficient of the *q*th scatterer in the *m-n*th observation channel, and $R_{mn,q}\left(t_{p}\right)$ is the propagation distance of the signal in the *m-n*th channel of the *q*th scatterer. $p_{m}\left(t\right)$ denotes the point spread function of $s_{m}\left(\hat{t}\right)$. $\forall m\in \left\{1,2,\cdots ,M\right\},p_{m}\left(t\right)\approx p\left(t\right)$ where p(t) is the sinc function [15].

The position vector of the *q*th scatterer can be written as

(2) $$r_{q}\left(t_{p}\right)=r_{q}\left[cos\kappa_{q0}\sin\left(\theta_{q}^{0}+\phi \left(t_{p}\right)\right),cos\kappa_{q0}\cos\left(\theta_{q}^{0}+\phi \left(t_{p}\right)\right),sin\kappa_{q0}\right],$$

where $r_{q}$ is the distance of the scatterer from the fulcrum *O*, $\theta_{q}^{0}$ is the initial azimuth angle, $\kappa_{q0}$ is the elevation angle of the *q*th scatterer above the XOY plane, and $\phi (t_{p})$ is the rotation angle at slow time $t_{p}$ measured in clockwise. So, $\theta_{q}^{0}+\phi \left(t_{p}\right)$ is the real azimuth angle at $t_{p}$.

The position unit vectors of the *m*th transmitting sensor and the *n*th receiving sensor are denoted as $R_{0m}$ and $R_{0n}$:

(3) $$R_{0m}=R_{0m}\left[-\cos\psi_{m}\sin\zeta_{m},-\cos\psi_{m}\cos\zeta_{m},-\sin\psi_{m}\right];$$

(4) $$R_{0n}=R_{0n}\left[-\cos\psi_{n}\sin\zeta_{n},-\cos\psi_{m}\cos\zeta_{n},-\sin\psi_{n}\right],$$

where $R_{0m}$ and $R_{0n}$ are the distance between the fulcurm and the transmitting or receiving sensor, $\zeta_{n}$ ($\zeta_{m}$) is the angle between the projection of the line connecting the receiving sensor (the transmitting sensor) and the target fulcrum on the XOY plane and the positive direction of *Y*-axis, which is drawn in Figure 1. $\psi_{m}$ and $\psi_{n}$ are grazing angle of the transmitting and receiving sensors. For the sake of simplicity, the grazing angles of all sensors are assumed to be the same as $\psi_{0}$, which is reasonable when the sensors are not very far from each other and the target locates far away.

Therefore, under the far-field assumption, the propagation distance $R_{mn,q}\left(t_{p}\right)$ can be expressed approximately as

(5) $$\begin{array}{ccc} R_{mn,q}\left(t_{p}\right)\;\; & =\;\; & ∥R_{0m}\;-\;r_{q}\left(t_{p}\right)∥\;+\;∥R_{0n}\;-\;r_{q}\left(t_{p}\right)∥\\ & =\; & R_{0m}+R_{0n}-\left(\frac{R_{0m}}{R_{0m}}+\frac{R_{0n}}{R_{0n}}\right)r_{q}\left(t_{p}\right)\\ & =\; & 2(R_{m\;n}+r_{q}\;\cos\kappa_{q0}\cos\psi_{0}\cos\left(\theta_{q}^{0}\;+\;\phi \left(t_{p}\right)\;-\;\;\alpha_{m\;n}\right)\cos\beta_{m\;n}+\sin\psi_{0}\sin\kappa_{q0}), \end{array}$$

where $R_{mn}=\left(R_{0m}+R_{0n}\right)/2$, $\alpha_{mn}=\left(\zeta_{m}+\zeta_{n}\right)/2$, and $\beta_{mn}=\left(\zeta_{m}-\zeta_{n}\right)/2$ are the mean distance, mean angle, and the half difference angle of the *m-n*th transmitting/receiving channel pair, respectively [4].

The transmitting/receiving pair (m,n) can be regarded as the *i*th equivalent sensor. By setting $\alpha_{i}=\alpha_{mn}$ and $\beta_{i}=\beta_{mn}$ and neglecting the constant distance $R_{mn}$ under the assumption that the translation motion has been compensated, $R_{mn,q}\left(t_{p}\right)$ can be written as

(6) $$r_{iq}\left(t_{p}\right)=2r_{q}\left(\cos\kappa_{q0}\cos\psi_{0}\cos\left(\theta_{q}^{0}+\phi \left(t_{p}\right)-\alpha_{i}\right)\cos\beta_{i}+\sin\psi_{0}\sin\kappa_{q0}\right)$$

Therefore, the received signal of the *i*th equivalent sensor can be expressed as

(7) $$S_{i}\left(\hat{t},t_{p}\right)=\sum_{q=1}^{Q}\sigma_{i,q}p\left[\hat{t}-r_{iq}\left(t_{p}\right)/c\right]e^{-j\frac{2\pi }{\lambda }r_{iq}\left(t_{p}\right)}.$$

**Figure 2:**
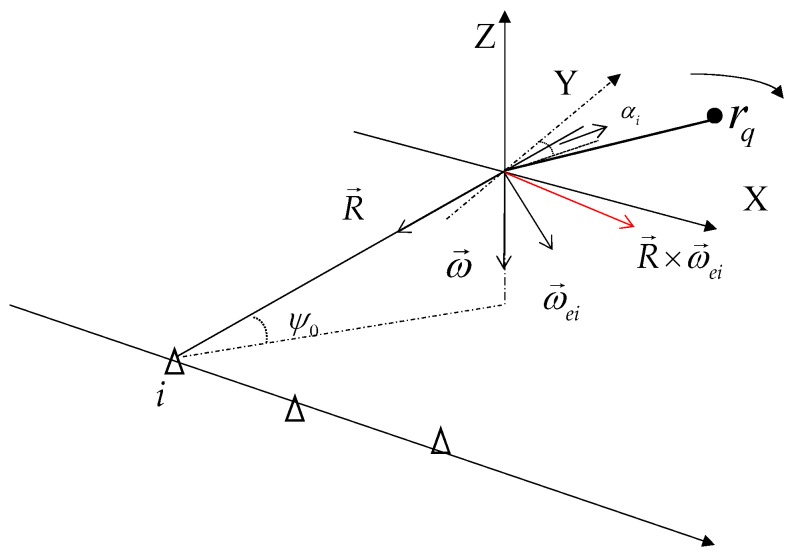
The *i*th IPP.

Due to the spatial separation of each equivalent sensor, the imaging projection plane (IPP) may not be the same. Since each equivalent sensor can be regarded as working independently, and the analysis of them are similar, we only use the analysis of the *i*th equivalent sensor to illustrate. As shown in Figure 2 , since the target rotates around the Z-axis, the rotation vector $\vec{\omega }$ is very simple as $\vec{\omega }=\left|\omega \right|\left[0,0,-1\right]$. ${\vec{R}}_{i}$ represents the range unit vector of the *i*th IPP and is pointed from the fulcrum *O* to the *i*th equivalent sensor.

In the ISAR imaging, the azimuth of the IPP is the cross-product of the range unit vector and the effective rotation vector ${\vec{\omega }}_{ei}$, so the red line in Figure 2 represents the direction of azimuth. The *i*th IPP is the plane containing ${\vec{R}}_{i}$ and ${\vec{R}}_{i}\times {\vec{\omega }}_{ei}$.

When the IPPs are not the same, neither are the subimages of different observation angles. In order to make all the subimages in the same plane, it needs to project them into the unified IPP [6]. Therefore, we assume that $\psi_{0}=0$, according to the rotation vector $\vec{\omega }$, the IPP is the XOY plane in Figure 1. However, we must declare that in the real scene, the radar sensors should be placed reasonably so that the IPPs can be approximated as the same plane. Once the radar formation does not meet the requirement, the method in this paper need some modification.

Based on the above assumption, the imaging of the target is the projection in that plane. In this paper, the IPP is the XOY plane in Figure 1. Although any value of $\alpha_{i}$ and $\beta_{i}$ can be set at this case, in reality, we still have to rationalize the radar and limit the value of $\psi_{0}$, $\alpha_{i}$ and $\beta_{i}$. At the same time, assuming that the target has been projected onto the XOY plane, then let $\kappa_{q0}=0$, so $r_{iq}$ can be re-expressed as

(8) $$r_{iq}\left(t_{p}\right)=2r_{q}\cos\left(\theta_{q}^{0}+\phi \left(t_{p}\right)-\alpha_{i}\right)\cos\beta_{i}$$

Equations (7) and (8) are the new echo expression, which will be analyzed to get the target’s image.

For the distributed ISAR system, the observation time is short and $\phi (t_{p})$ is small. By using approximations $\cos\phi (t_{p})\approx 1$ and $\sin\phi (t_{p})\approx 0$ in the range dimension, which are reasonable as the range error caused by these approximations is negigible compared with the range resolution, the compressed range of the *q*th scatterer can be expressed as

(9) $$r_{iq}=2r_{q}\cos\left(\theta_{q}^{0}-\alpha_{i}\right)\cos\beta_{i}.$$

While in the cross-range dimension, we use more accurate approximations $\sin\left(\phi \left(t_{p}\right)\right)\approx \phi \left(t_{p}\right)$, $\cos(\phi \left(t_{p}\right))\approx 1$. Based on these approximations, $S_{i}\left(\hat{t},t_{p}\right)$ can be rewritten as

(10) $$\begin{array}{cc} S_{i}\left(\hat{t},t_{p}\right) & =\sum_{q=1}^{Q}\sigma_{i,q}p\left[\hat{t}-r_{iq}/c\right]\\ & \cdot \exp\left[-j4\pi r_{q}\cos\left(\theta_{q}^{0}-\alpha_{i}\right)\cos\beta_{i}/\lambda \right]\\ & \cdot \exp\left[j4\pi r_{q}\sin\left(\theta_{q}^{0}-\alpha_{i}\right)\phi \left(t_{p}\right)\cos\beta_{i}/\lambda \right]. \end{array}$$

In Equation (10), the first exponent term is a constant related to the scattering point and transmitting/receiving channel, which has no effect on the imaging result. The cross-range imaging information is contained in the second exponential phase. For nonuniform rotation target, $\phi (t_{p})$ is a polynomial function and $S_{i}\left(\hat{t},t_{p}\right)$ is a polynomial phase signal (PPS). If the cross-range compression is achieved by using the FT, the second order terms (corresponding to the rotational acceleration) or even higher order terms (corresponding to the high order rotational motion) of $\phi (t_{p})$ will make the image blurred in the cross-range dimension. To avoid this effect, instead of using FT-based imaging method, the MFT is used here for cross-range compression. The MFT is a generalization of the FT and can effectively deal with the PPS.

## 3. Image Fusion of Nonuniform Rotation Target

### 3.1. Single ISAR Imaging by Matching Fourier Transform

The MFT can focus signals with nonlinear phase changes [16,17]. Consider a continuous signal $f\left(t\right)=\sum A_{i}e^{-j\omega_{i}\varphi \left(t\right)}$ with observation time $\left[0,T_{a}\right]$, where φ(t) is the frequency modulation function. If φ(t) is monotonic bounded and φ(0)=0, the MFT of f(t) can be achieved by

(11) $$F\left(\omega \right)=\int_{0}^{T_{a}}f\left(t\right)e^{-j\omega \varphi (t)}d\varphi \left(t\right),$$

where ω is the MFT frequency.

As aforementioned, for a nonuniform rotation target, $\phi (t_{p})$ is a polynomial function. We use $\omega_{0}$, $\omega_{1}$, ⋯ to represent the different order components of the rotational angular velocity, $\omega_{0}$ represents the uniform rotational component, $\omega_{1}$ represents the first-order rotational acceleration and so on. Therefore, the rotation angle $\phi (t_{p})$ can be expanded as

(12) ϕ(tp)=∑d=1ωd−1tpdωd−1tpdd!d!=w0(∑d=1ed−1tpded−1tpdd!d!)=w0ϑ(tp),

where $e_{d-1}=w_{d-1}/w_{0}$ ($e_{0}=1$). $\vartheta (t_{p})$ is determined by the target rotation characteristics and is the same for all scatterers.

Substituting (12) into (10) and neglecting the first constant term in (10), the cross-range signal in one range bin can be rewritten as

(13) $$S_{i}\left(t_{p}\right)=\sum_{q=1}^{Q}\sigma_{i,q}[\exp[j4\pi {x^{\prime }}_{iq}\omega_{0}\vartheta \left(t_{p}\right)/\lambda ],$$

and

(14) $${x^{\prime }}_{iq}=r_{q}\sin\left(\theta_{q}^{0}-\alpha_{i}\right)\cos\beta_{i}$$

is the equivalent cross-range position.

It is clear that (13) is the sum of plurality of signals with the same frequency modulation function $\vartheta (t_{p})$ and $\vartheta (t_{p})$ is the monotonic bounded function with ϑ(0)=0, which meets the definition of f(t). Thus, with the estimation of $e_{d-1}$, the MFT can be applied to $S_{i}\left(t_{p}\right)$. *T* is defined as the observation time, so the MFT expression is:

(15) $$S_{i}\left(\omega \right)=\int_{0}^{T}\sum_{q=1}^{nq}\sigma_{i,q}\exp\left\{j\left(4\pi {x^{\prime }}_{iq}\omega_{0}/\lambda +\omega \right)\vartheta \left(t_{p}\right)\right\}d\vartheta \left(t_{p}\right).$$

Using the linear properties of the MFT and letting fd=ωω2π2π, we can get

(16) $$\begin{array}{cc} S_{i}\left(f_{d}\right)= & \sum_{q=1}^{nq}\sigma_{i,q}\vartheta \left(T\right)\mathrm{sinc}\left[\vartheta \left(T\right)\left(f_{d}-2{x^{\prime }}_{iq}\omega_{0}/\lambda \right)\right]\\ & \exp\{-j\pi \vartheta \left(T\right)\left(f_{d}+2{x^{\prime }}_{iq}\omega_{0}/\lambda \right)\}. \end{array}$$

$S_{i}\left(f_{d}\right)$ is a set of narrow sinc pulses after the MFT, and the equivalent cross-range position of the scattering point can be calculated from the peak position $f_{dq}$ of the pulse with the formula ${x^{\prime }}_{iq}=f_{dq}\lambda /2\omega_{0}$. Thus, the scatters are all focused both in range and cross range dimension. The width of the sinc pulse is 1/ϑ(T), and the equivalent cross-range resolution is $\rho_{e}=\lambda /2\omega_{0}\vartheta \left(T\right)=\lambda /2\phi \left(T\right)$. Consider two scatterers in the same range bin, i.e., their coordinate difference is $\left(\Delta x,0\right)$. By expanding ${x^{\prime }}_{iq}$ as $\left(x_{q0}\cos\alpha_{i}-y_{q0}\sin\alpha_{i}\right)\cos\beta_{i}$ ( where $x_{q0}=r_{q}\sin\theta_{q}^{0}$, $y_{q0}=r_{q}\cos\theta_{q}^{0}$), it is easy to get that the distance of these two scatterers in the image is $\Delta x\cos\alpha_{i}\cos\beta_{i}$. Thus, in order to resolve these two scatterers, the following condition should be satisfied

(17) $$\Delta x\cos\alpha_{i}\cos\beta_{i}>\lambda /2\phi \left(T\right).$$

Obviously, after obtaining the estimation of the ratio $e_{d-1}$ of the target rotation parameters, MFT is very succinct and can directly obtain the azimuthal focused results for all range bins. This article assumes that $e_{d-1}$ has been estimated by other estimation methods. When the target’s maneuvering is not severe, the PPS model degrades into an LFM signal. Accoding to previous studies, fractional Fourier transform (FrFT) [18], Radon-Wigner transform [19], adaptive Chirplet decomposition [20] and centroid frequency-chirp rate distribution(CFCRD) [21] are effective approaches for parameter estimation of LFM signals. When the target’s maneuveirng is severe, several algorithms for cubic coefficient estimation can be retrieved, such as higher-order ambiguity function-integrated cubic phase function [22], scaled Fourier transform [23], keystone time-chirp rate distribution (KTCRD) [24]. Meanwhile, the rotational parameters can also be estimated according to the echoes from multi-channels [1,25,26].

### 3.2. Subimage Fusion

By applying the range compression and the MFT to the echoes of all transmitting/receiving channels, all subimages can be obtained. Due to the different values of RCS in different transmitting/receiving channels, the quality of these subimages has large fluctuations. In some subimages, the scatterers can be clearly distinguished. While in other subimages, some scatterers are submerged in noise. To obtain a stable imaging result, accumulation of all subimages is performed in the image domain.

#### 3.2.1. Subimage Resampling

For subsequent image processing, the first operation is to equalize the sampling grid in the range and cross-range dimension. Denote the sampling grid of the original image as $\left(\Delta l_{s},\Delta l_{a}\right)$, where $\Delta l_{s}$ and $\Delta l_{a}$ are the range sampling interval and the cross-range sampling interval:

(18) $$\Delta l_{s}=c/2f_{s};$$

(19) $$\Delta l_{a}=\lambda PRF/2wN_{f},$$

where $f_{s}$ is the sampling frequency in range dimension, PRF is the pulse repetition frequency, and $N_{f}$ is the number of points of the MFT.

Denote the sampling grid of the image after resampling as $\left(\Delta l_{s}^{\prime },\Delta l_{a}^{\prime }\right)$. According to the relation between $\Delta l_{s}$ and $\Delta l_{a}$, in order to make $\Delta l_{s}^{\prime }=\Delta l_{a}^{\prime }$, the original image can be up-sampled or down sampled in the range dimension or cross-range dimension.

#### 3.2.2. Subimage Scaling and Rotation

Use ${y^{\prime }}_{iq}$ to reformulate the range compression position $r_{iq}$ of the *q*th scatterer in the *i*th equivalent sensor, according to the expression of $r_{iq}$, ${y^{\prime }}_{iq}$ can be expressed as

(20) $$\begin{array}{cc} {y^{\prime }}_{iq} & =r_{q}\cos\left(\theta_{q}^{0}-\alpha_{i}\right)\cos\beta_{i}\\ & =\left(x_{q0}\sin\alpha_{i}+y_{q0}\cos\alpha_{i}\right)\cos\beta_{i}. \end{array}$$

After the MFT, the cross-range compression position can be expressed as

(21) $$\begin{array}{cc} {x^{\prime }}_{iq} & =r_{q}\sin\left(\theta_{q}^{0}-\alpha_{i}\right)\cos\beta_{i}\\ & =\left(x_{q0}\cos\alpha_{i}-y_{q0}\sin\alpha_{i}\right)\cos\beta_{i}. \end{array}$$

**Figure 3:**
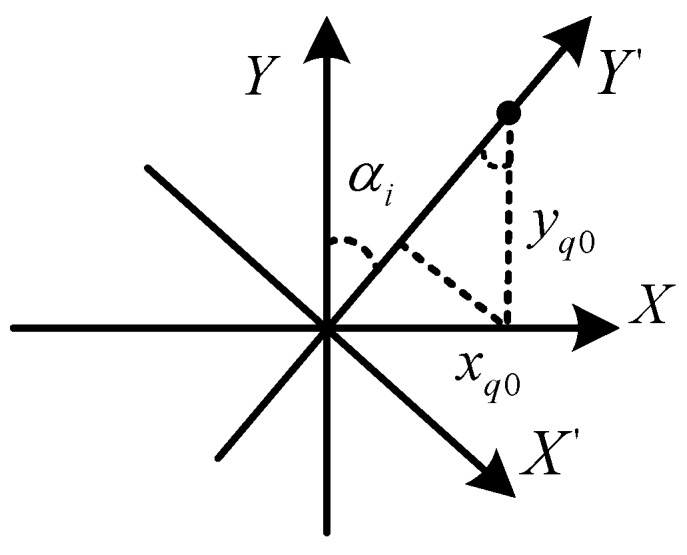
The rotation relationship of subimage.

Therefore, the imaging position of a scatterer $\left(x_{q0},y_{q0}\right)$ in the *i*th subimage is $\left(x_{iq}^{\prime },y_{iq}^{\prime }\right)$. $\left(x_{iq}^{\prime },y_{iq}^{\prime }\right)$ is related to $\alpha_{i}$ and $\beta_{i}$, which are different from a subimage to another subimage. To combine all subimages, the influence of $\alpha_{i}$, $\beta_{i}$ must be removed so that the imaging positions of the same scatterer in different subimages are aligned. By examing (20) and (21), the relationship between $\left(x_{q0},y_{q0}\right)$ and $\left(x_{iq}^{\prime },y_{iq}^{\prime }\right)$ can be represented by the rotation and scaling of the coordinate system as shown in Figure 3, which can be expressed as

(22) $$\left[\begin{array}{c} x_{iq}^{{}^{\prime }}\\ y_{iq}^{{}^{\prime }}\\ 1 \end{array}\right]=\cos\beta_{i}\left[\begin{array}{ccc} \cos\alpha_{i} & -\sin\alpha_{i} & 0\\ \sin\alpha_{i} & \cos\alpha_{i} & 0\\ 0 & 0 & 1 \end{array}\right]\left[\begin{array}{c} x_{q0}\\ y_{q0}\\ 1 \end{array}\right].$$

Thus, the alignment of all subimages can be achieved by applying an inverse scaling transform and an inverse rotation transform. The image transform is carried out in the homogeneous coordinate system. Assume the subimage $I_{i}$ has $G_{pix}$ pixels where $G_{pix}=L\times K$ (*L*, *K* means the sampling length in range and cross-range dimension). The homogeneous coordinate of the (l,k) pixel is represented as $i_{ilk}={\left[l,k,1\right]}^{T}$. By stacking the column vectors $\{i_{ilk}\},l=1,2,\cdots ,L,k=1,2,\cdots ,K$ in the row dimension, the coordinates of $I_{i}$ is converted into a $3\times G_{pix}$ dimensional matrix. Therefore, the corrected subimage matrix ${I^{\prime }}_{i}$ can be expressed as $I_{i}^{\prime }=T_{r}T_{s}I_{i}$ [27], where the scaling matrix $T_{s}$ and rotation matrix $T_{r}$ are expressed as

(23) $$T_{s}=\left[\begin{array}{ccc} \cos\beta_{i}^{-1} & 0 & 0\\ 0 & \cos\beta_{i}^{-1} & 0\\ 0 & 0 & 1 \end{array}\right],$$

(24) $$T_{r}=\left[\begin{array}{ccc} \cos\alpha_{i} & \sin\alpha_{i} & 0\\ -\sin\alpha_{i} & \cos\alpha_{i} & 0\\ 0 & 0 & 1 \end{array}\right].$$

After scaling and rotation, the coordinates of some pixel points of the subimage may not be integers, and the values are not defined for such coordinates which should be estimated from their neighbors. To achieve this, the bilinear interpolation algorithm for image is adopted here.

#### 3.2.3. Subimage Fusion

After the above processing, each scattering point is located at the same pixel in all subimages. Therefore, the final image can be obtained by the accumulation of all the subimages. The weight coefficients of subimages are calculated adaptively according to the image qualities of different subimages. Herein, the entropy is used as a quality metric.

The entropy of subimage $I_{i}$ [28,29,30] is defined as

(25) $$Ep=-\sum_{l=1}^{L}\sum_{k=1}^{K}D(l,k)\ln[D(l,k)],$$

where $D\left(l,k\right)=\left|d(l,k)\right|/\sum_{l=1}^{L}\sum_{k=1}^{K}\left|d(l,k)\right|$, d(l,k) is the value of the pixel (l,k). The image entropy reflects the sharpness of the image, and the image with a small entropy value is clearer. Therefore, the image with a smaller entropy value is given more weight coefficients. The final fusion image can be accumulated by

(26) $$I_{f}=\sum_{i=1}^{I}I_{i}^{{}^{\prime }}/Ep_{i}.$$

**Figure 4:**
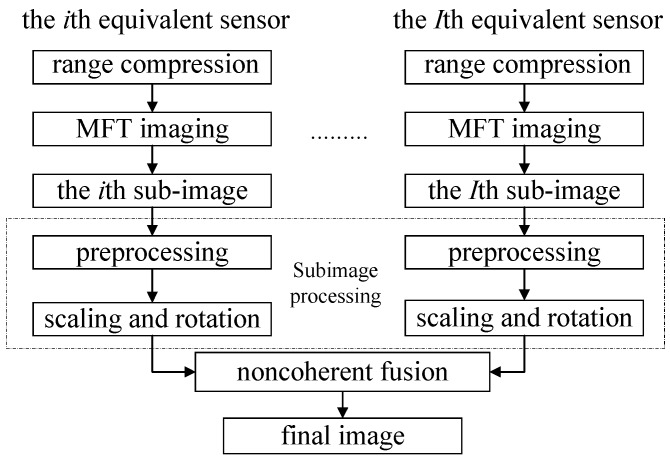
The complete processing chain.

Figure 4 shows the complete processing chain. Assume the translation motion of the target has been compensated, and after range compression and neglecting the range migration, the MFT is used for each equivalent channel’s echo to obtain the subimage. After the preprocessing, scaling and rotating of all subimages, the fusion image can be acquired by accumulating of all the subimages with coefficients determined by the subimages entropies.

## 4. Simulation and Experimental Results

In this section, simulations are conducted to demonstrate the effectiveness of the proposed method firstly. The distributed ISAR system are composed of four transmitting sensors and four receiving sensors (i.e., M=4, N=4). Therefore, sixteen equivalent sensors can be obtained.

### 4.1. Target Model and Echo Analysis

**Figure 5:**
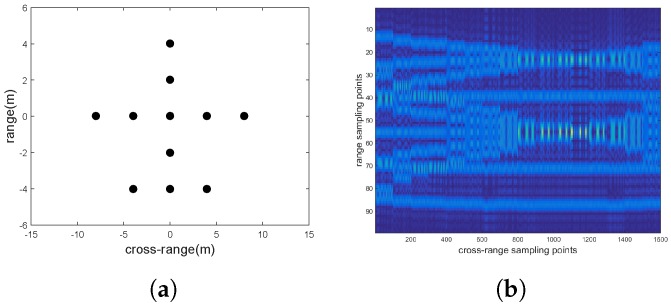
(**a**) Target model; (**b**) Echoes of all equivalent sensors.

The transmitting signal is a set of orthogonal signals with the same center frequency (10 GHz) and the same bandwidth (300 MHz) which can achieve a range resolution of 0.5 m. Assume the target rotates nonuniformly with an angular speed of 0.01 rad/s and an acceleration rate of $0.04\;\mathrm{rad}/s^{2}$, so we have $e_{1}$ is 4. The accumulation time is 0.5 s to achieve a cross-range resolution of 1.5 m. The target is composed of eleven scatterers (see Figure 5a) which are isotropic and independent with each other and obey the Swerling I distribution model. Under this model, each scatterer has the same RCS value in the same observation channel, but it is distributed identically across all channels. Setting the noise power to $\varsigma_{n}^{2}$, for a given signal to noise ratio (SNR), the signal power $\varsigma_{s}^{2}$ can be calculated by $\varsigma_{s}^{2}=\varsigma_{n}^{2}\cdot 10^{SNR/10}$. *I* random numbers $\sigma_{i}$ that satisfy the Rayleigh distribution with $\varsigma_{s}$ as the standard deviation parameter are generated. Then the RCS value of the *i*th observation channel is set to $\sigma_{i}$ [9].

Figure 5b shows the range compressed echoes of all sixteen equivalent senors. Each equivalent sensor has 101 slow time sampling points, and there are 16 pieces of echo data. According to (20), the range compression position of each scatterer is related to $\alpha_{i}$ and $\beta_{i}$. Since the $\alpha_{i}$ and $\beta_{i}$ are different from each other, the cross-range signal of each scatterer point spreads over different range bins and there are range jump between different equivalent sensors. Therefore, the observation angle in one range bin cannot be increased by directly splicing the cross-range signal from different equivalent sensors.

As the RCS fluctuation of high-maneuvering target and the nonuniform rotation are the main problem that affects the image quality, we mainly consider overcoming these problems by imaging the nonuniform rotating target from multi-channels.

### 4.2. Comparision of FT and MFT

**Figure 6:**
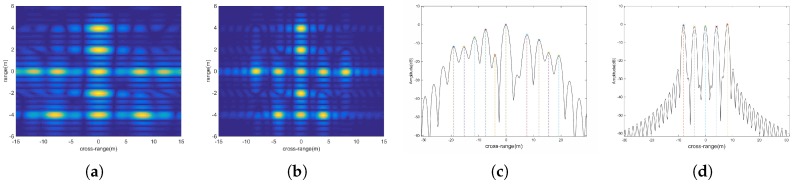
(**a**) Subimage by FT; (**b**) Subimage by MFT; (**c**) The profile of one range bin in (**a**); (**d**) The profile of one range bin in (**b**).

Assume we have already estimated the ratio of rotation parameters by parametric estimation methods [18,19,20,21,22,23,24], or we can estimate the rotation parameters $w_{0}$, $w_{1}$ [1,25,26] to calculate $e_{1}$. Figure 6a,b show the subimages (without noise) of the twelfth equivalent channel with mean angle $\alpha_{i}=0^{{}^{\circ}}$ and half difference angle $\beta_{i}=18^{{}^{\circ}}$, obtained from the FT and the MFT, respectively.

Figure 6c,d are the cross-range profiles of the range bin of (a) and (b) at range 0m. There are 5 scatterers in the range bin. It is evident that the MFT has better performance than the FT when dealing with nonuniform rotation target. Without a prior knowledge, it is impossible to recognize the 5 scatterers in Figure 6a. Compared with Figure 6b, the scatterers in Figure 6a have wider mainlobe and the power of their sidelobes are comparable with that of the mainlobes. Thus, the resolution of FT in this case is limited and it is easy subject to the sidelobe interference. Conversely, the cross-range profile obtained by the MFT is easily recognized and has lower sidelobe (about −25dB).

What needs to be pointed out here is that when the estimation of $\vartheta (t_{p})$ in (13) is inaccurate, there will be errors in the MFT results. However, as long as the error of $\vartheta (t_{p})$ is not too large, acceptable results can be still obtained by applying MFT.

### 4.3. Subimage Fuison

**Figure 7:**
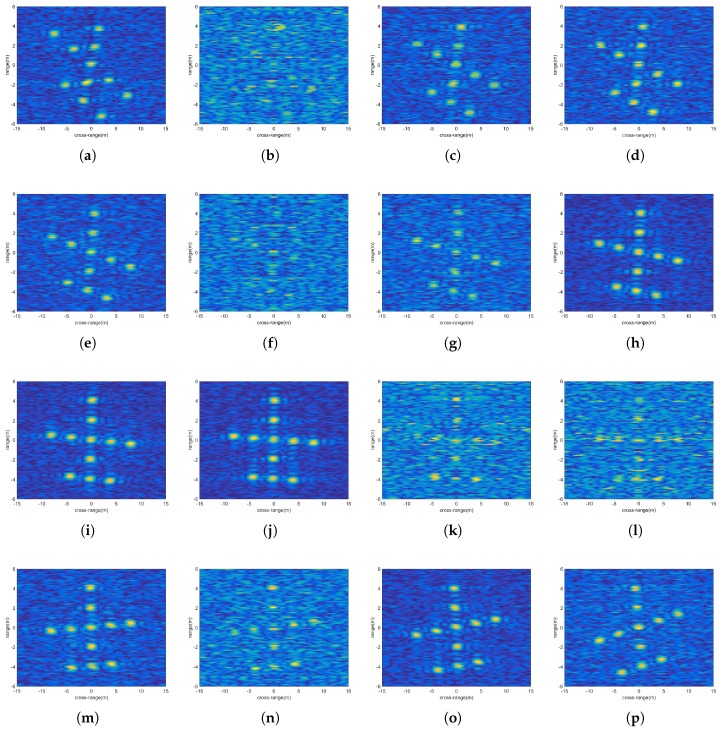
(**a**–**p**) Subimages of sixteen observation angles.

After applying MFT to range compressed echoes of all observation channels, the target images from different observation angles can be obtained. Figure 7 are the subimages from all the observation angles (SNR = 8 dB, $\varsigma_{n}^{2}=4$), and each subimage has a different degree of scaling and rotation.

**Figure 8:**
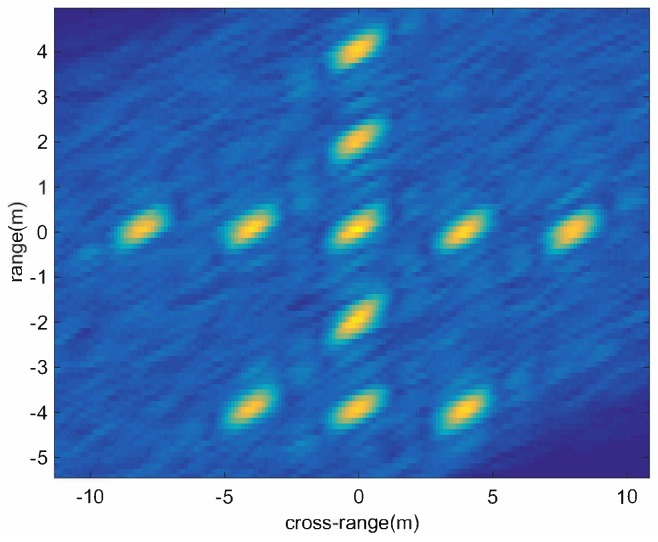
The fusion image.

Due to the RCS fluctuation, in some subimages (like (h),(i),(j)), the scattering points can be easily identified, while this is not true for other subimages (like (b),(k),(l)). The image quality of the corrected subimages can be judged directly from each subimage, and they are not drawn here. Figure 8 is the fusion image, in which the scattering points can be more clearly recognized. Table 1 shows the entropies of all subimages and the final fusion image. We can see that the final fusion image has the smallest entropy and the best image quality.

**Figure 9:**
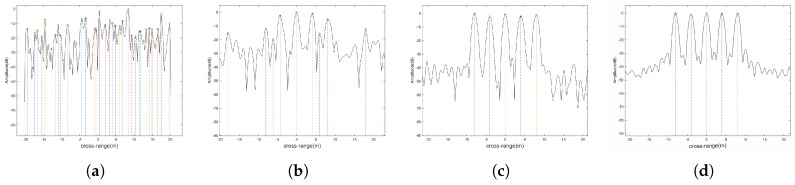
The profile of one range bin in (**a**–**c**) Three corrected subimages; (**d**) the fusion image .

To further analyze the performance of image fusion, the cross-range profiles of the range bin at range 0m are drawn. Three corrected subimages are selected. Figure 9a–c represents the cross-range profiles of corrected subimages of (b),(n),(o) in Figure 7. The noise of Figure 9a is very large, and the positions of the scattering points are completely indistinguishable. The scatterer positions in Figure 9b,c can be distinguished, but the noise fluctuations in (b) are still very obvious. From the fusion image in Figure 9d, it can be seen that the noise power has been reduced significantly and the positions of scatterers are clearly distinguishable. Although the non-coherent fusion of the subimages does not improve the image resolution, the image quality after the fusion is indeed improved.

### 4.4. Application to Live Data

**Figure 10:**
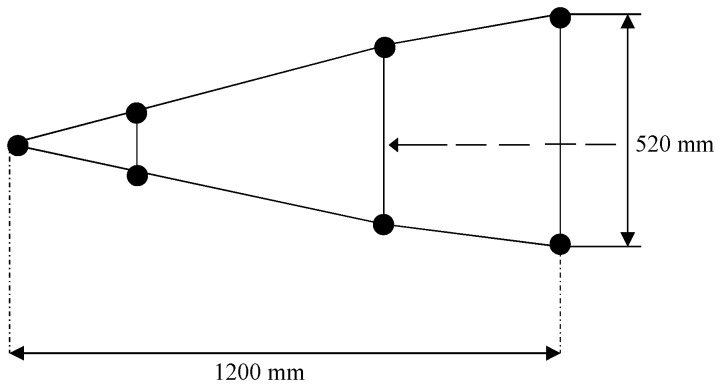
The size and the shape of the conical target.

The aforementioned technique has been applied to the measured data of the conical target in the microwave anechoic chamber. The target is placed in the center of the turntable and remain motionless. The radar rotates around the target with the turntable and emits 8 GHz to 10 GHz stepped frequency modulated (SFM) signal with step of 20 MHz. The size and the shape of the target are shown in Figure 10 and the initial radar LOS is indicated by the dotted line. The PRF is 400 Hz. We simulate multiple equivalent sensors simultaneously observe the target by selecting radar echoes from different observation angles. In addition, we non-uniformly select the slow time sampling points to simulate nonuniform rotation of the target. Meanwhile, we add different levels of noise to the echoes of different observation channels to simulate the fluctuation of the background noise.

**Figure 11:**
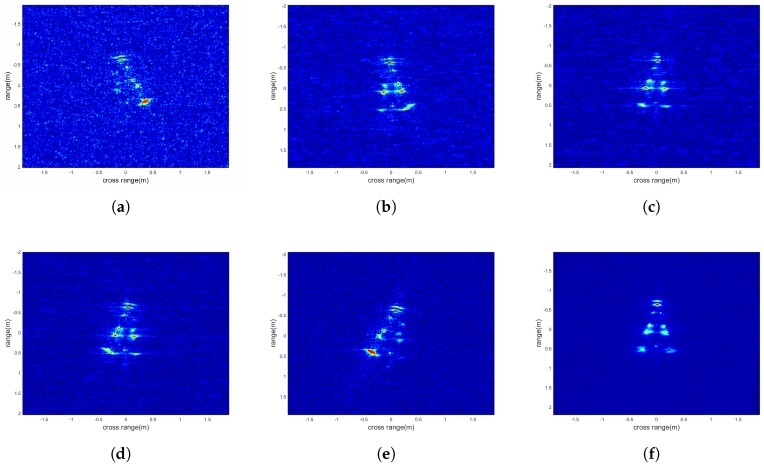
The conical target (**a**–**e**) subimages from different observation angles; (**f**) The fusion image.

After range compression and MFT, we obtain imaging results from five different observation angles, that is $\left[-10^{{}^{\circ}},-5^{{}^{\circ}},0^{{}^{\circ}},5^{{}^{\circ}},10^{{}^{\circ}}\right]$. Figure 11a–e show the target with different rotation angles (since the half-difference angle of each observation angle is zero in this experiment, there is no scaling). Meanwhile, it also shows that in some observation angles, due to the mutual obstructions between scatterers, not all scatterers can be identified. Figure 11f is the non-coherent fused image, according to Table 2, the fused image has the smallest entropy and makes the shape of the target clearly visible from noise.

## 5. Conclusions

The distributed ISAR technique can utilize the data acquired from multiple observation angles. In this paper, a method which combines the distributed ISAR technique and the MFT is proposed to obtain the image of a high-maneuvering target. Two main problems, i.e., the RCS fluctuation and nonuniform rotation of high-maneuvering target, are solved.

In this paper, we assume that all IPPs are the same, which simplifies the processing chain. It should be pointed out that in order to satisfy this requirement, the radar sensors should not be placed far apart from each other in real scene. Based on the assumption, the multiple channel echoes of nonuniform rotation target are acquired from different observation angles firstly. Secondly, using the MFT for all channel echoes can avoid the azimuthal defocusing problem caused by the FT and get well-focused subimages. At the same time, by estimating the rotation parameters once, the MFT can apply directly to all the range bins of all echoes from different observation channel, which is computational effective. Thirdly, after the processing and accumulating of all subimages, the final fusion image can be acquired. To reduce the influence of RCS fluctuations, the accumulation coefficients are determined adaptively according to the subimage entropies. The simulations and experiment show the effectiveness of the proposed method to overcome the RCS fluctuation and can result in a final image with improved quality.

Thus, the innovativeness and contribution of this article are:

- Based on the characteristics of the high-maneuvering target, the distributed ISAR technique is used to observe the target from multi-channels with different RCS values.
- The MFT is applied to the echo of each channel to acquire well-focused subimages, which is computational efficient compared with other imaging methods.
- Subimages fusion with adaptive coefficients calculated according to subimage entropies can effectively overcome the RCS fluctuations.

In this paper, the rotation around the Z-axis is considered only. However, in real scene, the target always has three-dimensional rotation which is more complicated. In this case, obtaining stable and recognized ISAR image is challenge. Meanwhile, the IPPs are assumed to be the same in this paper. If the radar formation is not strictly limited, this assumption does not hold. Therefore, the subimages from different observation channels cannot be accumulated directly. Further research is necessary to increase the applicability of the method in this paper. Another research area of interest is how to use the raw data of each channel to get a ISAR image with higher resolution when the target rotates nonuniformly.

## Figures and Tables

**Table 1:** The image entropies of subimages and final fusion image (SNR = 8 dB, $\varsigma_{n}^{2}=4$).

$E_{p1}$	$E_{p2}$	$E_{p3}$	$E_{p4}$	$E_{p5}$	$E_{p6}$
5.6934	6.8958	5.9686	5.9141	5.8311	6.8305
$E_{p7}$	$E_{p8}$	$E_{p9}$	$E_{p10}$	$E_{p11}$	$E_{p12}$
6.2674	5.4732	5.4162	5.3319	6.9425	6.8433
$E_{p13}$	$E_{p14}$	$E_{p15}$	$E_{p16}$	$E_{pfinal}$	
5.8797	6.7876	5.5706	6.0895	5.4518	

**Table 2:** The image entropies of subimages and fusion image of the conical target.

$E_{p1}$	$E_{p2}$	$E_{p3}$	$E_{p4}$	$E_{p5}$	$E_{pfinal}$
5.4906	5.0120	4.6289	4.5569	4.4304	4.1634
